# A critical role for IL-17RB signaling in HTLV-1 tax-induced NF-κB activation and T-cell transformation

**DOI:** 10.1186/1742-4690-12-S1-P52

**Published:** 2015-08-28

**Authors:** Alfonso Lavorgna, Masao Matsuoka, Edward W Harhaj

**Affiliations:** 1 Department of Oncology, Sidney Kimmel Comprehensive Cancer Center, Johns Hopkins School of Medicine, Baltimore, Maryland, USA; 2 Laboratory of Virus Control, Institute for Virus Research, Kyoto University, Kyoto, Japan

## 

Human T-cell leukemia virus type 1 (HTLV-1) infection is linked to the development of adult T-cell leukemia (ATL) and the neuroinflammatory disease HTLV-1 associated myelopathy/tropical spastic paraparesis (HAM/TSP). The HTLV-1 Tax protein functions as a potent viral oncogene that constitutively activates the NF-κB transcription factor to transform T cells; however, the underlying mechanisms remain obscure. Here, using next-generation RNA sequencing we identified the IL-25 receptor subunit IL-17RB as an aberrantly over expressed gene in HTLV-1 immortalized T cells. Tax induced the expression of IL-17RB in an IκB kinase (IKK) and NF-κB-dependent manner. Remarkably, Tax activation of the canonical NF-κB pathway in T cells was critically dependent on IL-17RB expression. IL-17RB and IL-25 were required for HTLV-1-induced immortalization of primary T cells, and the constitutive NF-κB activation and survival of HTLV-1 transformed T cells. IL-9 was identified as an important downstream target gene of the IL-17RB pathway that drives the proliferation of HTLV-1 transformed cells. Furthermore, IL-17RB was over expressed in leukemic cells from a subset of ATL patients and also regulated NF-κB activation in some, but not all, Tax-negative ATL cell lines. Together, our results support a model whereby Tax instigates an IL-17RB-NF-κB feed-forward autocrine loop that is obligatory for HTLV-1 leukemogenesis.

